# Trans biliary proximal and distal coil embolization of an arteriobiliary fistula: report of a case and review of literature

**DOI:** 10.1186/s42155-018-0046-9

**Published:** 2019-01-04

**Authors:** Faris Galambo, Majid Maybody

**Affiliations:** 1 Rush University Medical Center, 1653 W Congress Pkwy, Chicago, IL 60612 USA; 2 Memorial Sloan Kettering Cancer Center, 1275 York Avenue, New York, NY 10065 USA

**Keywords:** Arterial injury, Percutaneous biliary drainage, Peri catheter bleeding, Hemobilia, Retrograde arterial embolization

## Abstract

**Background:**

Hepatic arterial injury is an uncommon complication of percutaneous transhepatic biliary drainage interventions that commonly presents with hemobilia and peri catheter hemorrhage. It is classically managed with antegrade trans arterial embolization. However, this approach may not be possible due to altered anatomy and alternative techniques need to be considered. We report a case of an arteriobiliary fistula which was successfully coil embolized both distal and proximal to the lesion using a trans biliary approach. This is the first report of such method and interventionalists should be aware of this option. The literature is reviewed.

**Case presentation:**

We report a case of a 49-year-old male with advanced colorectal cancer presented with cholangitis. His duodenal anatomy precludes endoscopic intervention, so he underwent percutaneous biliary drainage complicated by intractable hemobilia and pericatheter bleeding. Hepatic arterial anatomy evaluated by two catheter angiographies was shown to be isolated at multiple levels by tumors and prohibited antegrade access of bleeding artery for embolization. Sheath cholangiography revealed an arteriobiliary fistula involving left hepatic arterial branches. The arterial injury was successfully treated by coil embolization distal and proximal to the lesion via a retrograde trans biliary approach, with complete resolution of hemobilia.

**Conclusion:**

Trans biliary proximal and distal coil embolization is a newly reported approach for treating biliary hemorrhage when traditional antegrade arterial embolization is not feasible due to preclusive anatomic factors. Interventionalists should be familiar with this management option.

## Background

Hepatic arterial injury is an uncommon complication of percutaneous biliary drainage interventions (Saad et al. 2008). When symptoms of hemorrhage into the biliary tree such as pain, hemobilia, peri catheter hemorrhage and upper gastrointestinal bleeding persist despite conservative management, other interventions are indicated. These include upsizing of the biliary drainage catheter and arteriography/embolization. The angiographic manifestations of hepatic arterial injury include arteriobiliary or arterioportal fistula, pseudoaneurysm, extravasation and focal arterial caliber irregularity at the site of indwelling catheter. These findings may be obscured by the indwelling catheter and the contrast in the biliary ducts.

Sometimes antegrade angiography is not possible due to challenging anatomy and other techniques are required to control bleeding. We describe a case of intractable hemobilia from biliary drainage where altered anatomy by tumors precluded antegrade endovascular treatment. Cannulation of the injured artery via the biliary access site made distal and proximal embolization possible. Interventionalists should be aware of this option of managing biliary hemorrhage. Literature is reviewed.

## Case presentation

**Fig. 1:**
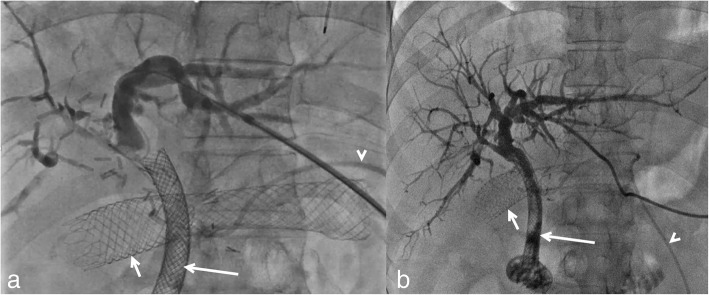
Initial cholangiogram during left IEBD catheter placement. The CBD stent (long arrow) is obstructed (**a**). There was no isolation of ducts (**b**). The proximal duodenal stent (short arrow) and catheter of HAIP (arrowhead) are evident

**Fig. 2:**
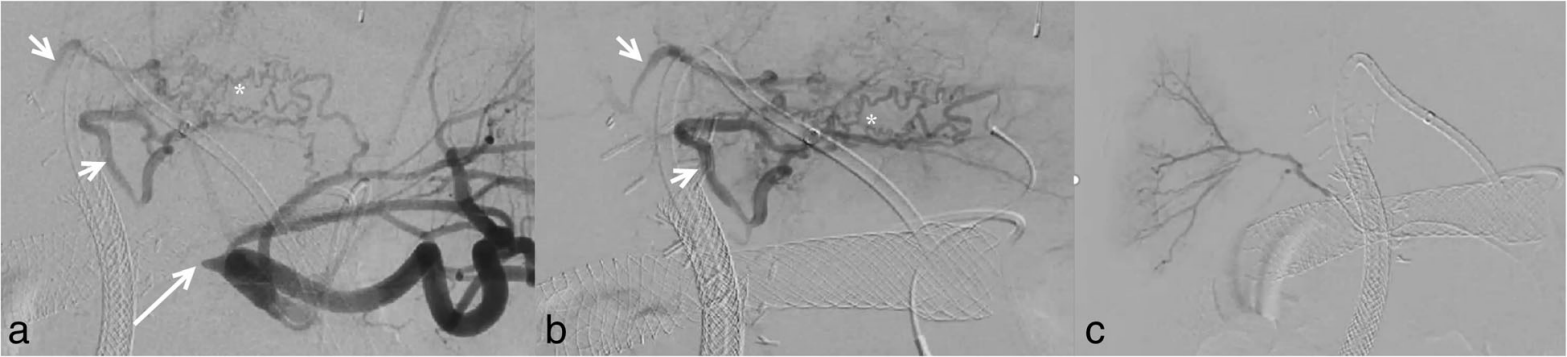
Hepatic arteriography shows (**a** and **b**) obstructed common hepatic artery (long arrow) and segment 2 and 3 hepatic arteries (short arrows) recanalized through small tortuous collaterals (asterisk) via left gastric artery. Selective arteriogram via a collateral vessel from the left gastric artery (**b**). Hepatic arteriography beyond obstructed common hepatic artery only shows a segmental right hepatic artery branch (**c**)

A 49-year-old male with metastatic colon adenocarcinoma presented with several days of fever, nausea, vomiting, jaundice and hyperbilirubinemia. His past medical history includes right hemicolectomy, right adrenalectomy, partial right hepatectomy and hepatic arterial infusion pump (HAIP) placement 4 years ago. He had received systemic and hepatic arterial pump chemotherapy. Six months prior to this admission he underwent endoscopic placement of two metallic stents across the proximal duodenal obstruction and common bile duct (CBD) obstruction from infiltrative metastases. Computed tomography (CT) scan of the abdomen showed bilobar biliary ductal dilatation due to stent occlusion. Portal vein was patent. Endoscopic biliary drainage failed as the CBD stent could not be accessed due to the presence of duodenal stent. Percutaneous biliary drainage was requested. Informed consent was obtained for all interventions. Cholangiography confirmed obstruction of the CBD stent and an internal-external biliary drainage (IEBD) catheter was placed via a segment 3 duct (Fig. 1). Needle access to segment 3 duct was performed under ultrasound guidance. The patient was readmitted 2 days following discharge due to chills, bacteremia, persistent hyperbilirubinemia, right upper quadrant pain, hematochezia, and bleeding inside and around the IEBD catheter. Culture results from the implantable port showed *E. coli*, other enteric bacteria, yeast and candida similar to bile and peripheral blood samples confirming biliary source of infection. Patient remained afebrile on antibiotics. Intermittent peri catheter bleeding, hemobilia and hematochezia persisted. Antegrade visceral angiography was performed on post-operative day 9. This showed complete obstruction of the common hepatic artery and recanalization of the left hepatic artery via small tortuous collaterals from the left gastric artery. No significant supply was seen from the superior mesenteric artery. The segment 3 branch of the left hepatic artery could not be separated from the biliary catheter on any oblique views confirming it as the source of hemobilia. Retrograde cannulation of the left hepatic artery via the collaterals was not possible (Fig. 2). The IEBD catheter was upsized from 8.5F to 12F in attempt to tamponade the injured vessel. Peri catheter bleeding and hemobilia persisted and 5 days later, he underwent repeat hepatic angiography. The common hepatic arterial occlusion was crossed with a 2.4 French microcatheter and 0.018-in. hydrophilic guidewire coaxially. This demonstrated multi-level occlusion of the hepatic arterial branches. The left hepatic artery could not be cannulated antegradely or retrogradely (Fig. 2).

**Fig. 3:**
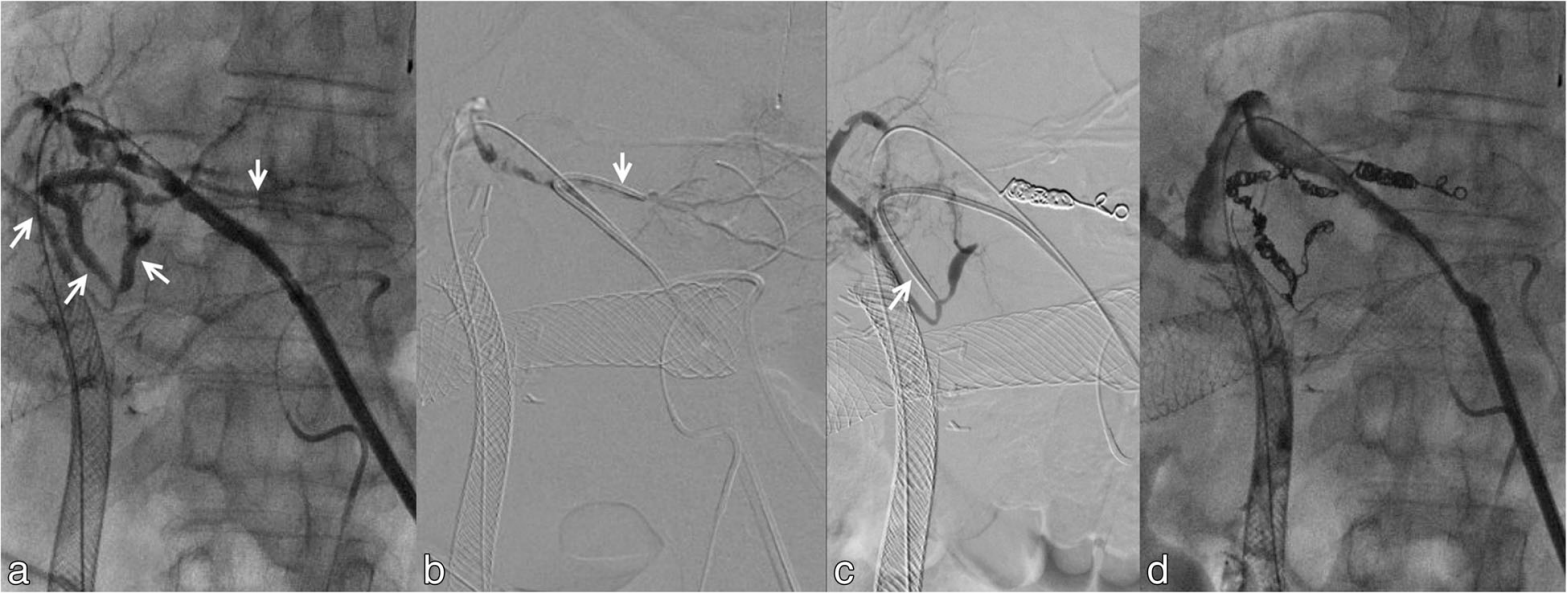
Sheath cholangiogram (**a**) shows lateral segment hepatic artery branches (short arrows). The segment 3 artery is cannulated both distal (**b**) and proximal (**c**) to the arteriobiliary fistula for coil embolization. Final sheath cholangiogram (**d**) shows resolution of flow in lateral segment arteries from the segment 3 bile duct

The indwelling IEBD catheter was exchanged over wire with a 10 French vascular sheath. Sheath cholangiography showed opacification of the segment 3 hepatic artery. This artery was successfully accessed via the vascular sheath both distal and proximal to its communication with the bile duct using a 5 French directional catheter and hydrophilic guidewire. Both areas of the artery were successfully embolized using a total of ten 0.035-in. and three 0.018-in. metallic coils of different lengths and diameters. Final sheath cholangiography showed no flow in the embolized artery (Fig. 3). The peri catheter hemorrhage and hemobilia resolved over the next 2 days. Secondary biliary stenting was performed successfully 6 weeks later. The patient remained asymptomatic and expired 2 months later due to progression of disease.

## Discussion and conclusions

Hemobilia results when a splanchnic vessel fistulizes with the intrahepatic or extrahepatic biliary tree. These most commonly result from iatrogenic trauma, though other causes include accidental trauma, gallstones, tumors, inflammation and vascular malformations (Green et al. 2001). Bleeding complications are seen in 2 to 3% of percutaneous transhepatic biliary drainage interventions and most commonly present as bleeding from the drain itself, though perihepatic and gastrointestinal bleeding may occur (Saad et al. 2008). Left-sided percutaneous biliary catheters are associated with greater risk of hepatic arterial injury compared with right-sided ones (Choi et al. 2011). Also challenging scenarios such as diverting biliary drainage when the ducts are decompressed due to leakage or biliary drainage in high bile duct obstruction when specific ducts need to be accessed are expected to have higher likelihood for complications including arterial injuries.

When hemobilia is noted, an appropriate initial step is to ensure proper catheter placement with all catheter side holes inserted within the biliary system. Reversible causes of hemobilia such as coagulopathy should also be assessed. Further workup is guided by history and typically involves esophagogastroduodenoscopy (EGD), CT imaging, and angiography (Green et al. 2001). Hepatic angiography can definitively demonstrate arterial injury including the presence of a fistula between the hepatic artery and bile ducts, portal or hepatic veins.

Antegrade trans arterial embolization (TAE) is a common first-line treatment for hemobilia when conservative management is insufficient, with a reported success rate of 80 to 100% (Saad et al. 2008; Green et al. 2001). Standard antegrade TAE may not be possible due to extreme hepatic vessel tortuosity and altered anatomy by surgery or disease and alternative approaches to embolization are required. In this patient, altered arterial anatomy may be secondary to metastases and prior HAIP chemotherapy. In such cases, the arterial system can be accessed via percutaneous transhepatic approach when no indwelling biliary catheter is present. It has been used for antegrade arterial coil embolization (Tamura et al. 2016), antegrade glue embolization (Venkatanarasimha et al. 2017) and retrograde stent grafting of a dissected common hepatic artery (Papadopoulos et al. 2014). Endoscopic placement of a covered stent in the bile duct across an arteriobiliary fistula can be performed (Kawakami et al. 2014).

In the presence of an indwelling biliary catheter, it can be used as an access to place a covered biliary stent across the arteriobiliary fistula (Tan and Kapoor 2008). Embolization of a right hepatic artery pseudoaneurysm (coil) and the proximal feeding branch (Gelfoam) via an indwelling biliary drain access is reported (Rosen and Rothberg 1982). Trans biliary focal coil embolization of an arteriobiliary fistula in the left hepatic artery when it was accidentally accessed through a right transhepatic approach is reported (Nakagawa et al. 1994).

In coil embolization of arterial injuries, the ideal technique is when the lesion is isolated from both antegrade and retrograde flow by distal and proximal embolization. In this report, a case of an arteriobiliary fistula is successfully coil embolized both distal and proximal to the lesion using a trans biliary approach. This is the first report of such approach.

In conclusion while arteriobiliary fistulae are typically treated with an anterograde endovascular approach, this may not always be possible. Knowledge of unconventional techniques for management of these complex scenarios is helpful to interventional radiologists. This report is intended to introduce a new technique and draw new attention to similar ones already reported.

## Abbreviations

CBD: Common bile duct
CT: Computed Tomography
EGD: Esophagogastroduodenoscopy
HAIP: Hepatic arterial infusion pump
IEBD: Internal external biliary drain
TAE: Trans arterial embolization
